# Determinants of health-related quality of life in people with Human Immunodeficiency Virus, failing first-line treatment in Africa

**DOI:** 10.1186/s12955-023-02179-x

**Published:** 2023-08-21

**Authors:** Tamlyn A. Rautenberg, Shu Kay Ng, Gavin George, Mahomed-Yunus S. Moosa, Suzanne M. McCluskey, Rebecca F. Gilbert, Selvan Pillay, Isaac Aturinda, Kevin L. Ard, Winnie R. Muyindike, Nicholas Musinguzi, Godfrey Masette, Melendhran Pillay, Pravi Moodley, Jaysingh Brijkumar, Rajesh T. Gandhi, Brent Johnson, Henry Sunpath, Mwebesa B. Bwana, Vincent C. Marconi, Mark J. Siedner

**Affiliations:** 1 School of Medicine and Dentistry, Centre for Applied Health Economics, Griffith University, Australia (Sir Samuel Griffith Centre N78 Room 2.34), Nathan campus, QLD 4111 Australia; 2 Menzies Health Institute Queensland, Southport, Australia; 3 Metro North Hospital and Health Service Queensland, Herston, Australia; 4 Health Economics and HIV Research Division, University of KwaZulu-Natal, Durban, South Africa; 5 Division of Social Medicine and Global Health, Lund University, Lund, Sweden; 6 College of Health Sciences, University of KwaZulu-Natal, Durban, South Africa; 7 Department of Medicine, Massachusetts General Hospital, Boston, MA USA; 8 Department of Medicine, Harvard Medical School, Boston, MA USA; 9 Faculty of Medicine, Mbarara University of Science and Technology, Mbarara, Uganda; 10 Africa Health Research Institute, KwaZulu-Natal, Durban, South Africa; 11 National Health Laboratory Service, Durban, South Africa; 12 Department of Biostatistics and Computation Biology, University of Rochester, Rochester, NY USA; 13 Department of Medicine, Emory University School of Medicine, Atlanta, GA USA; 14 Department of Global Health, Rollins School of Public Health, Atlanta, GA USA

**Keywords:** Virological failure, Health state utility values, EQ-5D, HIV, Health related quality-of-life, Two part regression

## Abstract

**Background:**

Antiretroviral treatment improves health related quality of life (HRQoL) of people with human immunodeficiency virus (PWH). However, one third initiating first-line treatment experience virological failure and the determinants of HRQoL in this key population are unknown. Our study aims to identify determinants of among PWH failing antiretroviral treatment in sub-Saharan Africa.

**Methods:**

We analysed data from a cohort of PWH having virological failure (> 1,000 copies/mL) on first-line ART in South Africa and Uganda. We measured HRQoL using the EuroQOL EQ-5D-3L and used a two-part regression model to obtain by-country analyses for South Africa and Uganda. The first part identifies risk factors that were associated with the likelihood of participants reporting perfect health (utility = 1) versus non-perfect health (utility < 1). The second part identifies risk factors that were associated with the EQ-5 L-3L utility scores for participants reporting non-perfect health. We performed sensitivity analyses to compare the results between the two-part model using tobit models and ordinary least squares regression.

**Results:**

In both countries, males were more likely to report perfect health and participants with at least one comorbidity were less likely to report perfect health. In South Africa, participants with side effects and in Uganda those with opportunistic infections were also less likely to report perfect health. In Uganda, participants with 100% ART adherence were more likely to report perfect health. In South Africa, high HIV viral load, experiencing ART side effects, and the presence of opportunistic infections were each associated with lower HRQoL, whereas participants with 100% ART adherence reported higher HRQoL. In Uganda participants with lower CD4 count had lower HRQoL.

**Conclusion:**

Markers of advanced disease (opportunistic infection, high viral load, low CD4), side effects, comorbidities and lack of ART adherence negatively impacted HRQoL for PWH experiencing virological failure.

**Trial registration:**

ClinicalTrials.gov: NCT02787499.

**Supplementary Information:**

The online version contains supplementary material available at 10.1186/s12955-023-02179-x.

## Highlights

What we already know:

As antiretroviral treatment (ART) access has expanded for people with HIV (PWH) and the condition has transitioned from a routinely fatal to chronic disease in sub-Saharan Africa, longer term health-related quality of life (HRQoL) has become the primary focus of care.

ART generally improves HRQoL, but up to one third of PWH initiating first-line ART will experience virological failure.

The gap:

The determinants of HRQoL for PWH suffering virologic failure in sub-Saharan Africa are unknown.

What our study adds:

Markers of advanced disease (opportunistic infection, high viral load, low CD4), side effects, comorbidities and lack of ART adherence negatively impacted HRQoL for PWH experiencing virological failure.

**Supplementary Information:**

The online version contains supplementary material available at 10.1186/s12955-023-02179-x.

## Background

Human immunodeficiency virus (HIV) has transitioned from a life-threatening to chronic, treatable condition [1, 2]. This shift has expanded the focus of HIV treatment programs, from reducing morbidity and mortality, to improving health-related quality of life (HRQoL) [3–5].

Evidence informs that antiretroviral therapy (ART) improves HRQoL, but one in three individuals who initiate therapy experience virological failure, and there is limited information on correlates of HRQoL for this population [5–10]. Measuring and understanding the determinants of HRQoL among people with virological failure are critical to ensuring that healthcare services deliver people-centred management to optimise patient outcomes. This is particularly relevant in Sub-Saharan Africa due to constrained healthcare resources, disproportionate disease burden and lower socio-economic status. In addition, evidence on determinants of HRQoL for people with HIV (PWH) is contradictory, some studies report higher HRQoL for male PWH while others report higher HRQoL for women PWH [11–17].

We analysed clinical characteristics and HRQoL data from PWH with virological failure in order to understand the factors associated with HRQoL among PWH experiencing virological failure and thereby advance clinical management strategies of this key patient population.

## Methods

### Study design

Data for this analysis were derived from the REVAMP clinical trial (NCT02787499). The design and primary results of the trial have been previously described [18, 19]. In summary, REVAMP was a pragmatic clinical trial, designed to investigate the impact of adopting resistance testing to improve management of virological failure for PWH on first-line ART in sub-Saharan Africa. We conducted the study in South Africa and Uganda, two lower-middle income countries with similar healthcare, socio-demographic and economic characteristics, where viral load testing was routine practice. All participants were 18 years or older and were on first-line nonnucleoside reverse transcriptase inhibitor-based ART for at least 5 months. All participants had virological failure on first-line ART measured by 2 successive HIV-1 plasma RNA viral load (VL) measurements > 1,000 copies/mL. Participants with known HIV drug resistance or prior exposure to protease inhibitors were excluded.

### Data collection and definitions

Individual participant data were collected at study enrolment and is the focus of this paper. To measure HRQoL we administered the EuroQOL EQ-5D-3L questionnaire and converted raw scores to health state utility values based on the general population time trade-off dataset for Zimbabwe as the most representative available dataset with HRQoL valuation data from the region [20]. CD4 + T-cell counts and VL measurements were abstracted from medical charts and divided into three (< 200; 200–499; ≥500 cells/uL) and two categories (≤ 50,000; >50,000 copies/mL), respectively [21]. Presence of comorbidities was defined as self-reporting any of the following comorbidities for the preceding 6-month study period: heart disease, diabetes, hypertension, kidney disease, lung disease, gastrointestinal illness, mental health illness, and other. Presence of an opportunistic infection was defined as self-reporting any of the following opportunistic infections: tuberculosis, extrapulmonary tuberculosis, cryptococcal meningitis, esophagitis, pneumonia, or Kaposi’s sarcoma. Adherence to ART was self-reported for the preceding six months. Participants reporting 100% adherence to ART were classified as adherers, and all other participants were classified as non-adherers (adherence < 100%).

### Statistical analyses

We performed by-country analyses for Uganda and South Africa. The health state utility values were captured on a scale of worst health (utility = 0) and perfect health (utility = 1). We analysed the baseline EQ-5D-3L data using a two-part regression model, which is widely used in health economics and health services research to account for the mass of ones in utility values (a ceiling effect of reported health) [22–26]. We considered and explored all variables measured at baseline which were: age measured in years, gender, ART duration (years), CD4 + T-cell counts, VL, ART adherence, presence of comorbidities, presence of opportunistic infection, and side effects. We defined low VL as ≤ 50,000 and high VL as > 50,000 copies/mL and low CD4 as < 200 cells/uL, medium CD4 between 200 and 499 cells/uL and high CD4 as ≥ 500 cells/uL. We included all variables in both models at the start and dropped non-significant variables (p > 0.05) from the final models.

Part I of the model uses a logistic regression to identify risk factors that were associated with the likelihood of all participants reporting the binary outcomes perfect health (utility = 1) versus non-perfect health (utility < 1). Part II of the model uses a generalised linear model with a gaussian distribution and an identity link function to identify risk factors that were associated with the EQ-5D-3L utility scores for participants reporting non-perfect health (utility < 1) [27].

We performed sensitivity analyses to compare the results between the two-part regression model using tobit (accounting for the distribution of utility data with an upper bound at 1), and ordinary least squares regression models. Interaction terms between risk factors were considered in all analyses. Model fit was compared based on the Bayesian information criterion. Statistical analysis was performed in STATA (SE 16.0, Stata Corp, College Station, TX).

### Ethical considerations

Approval for study procedures was obtained from Mbarara University of Science and Technology Research Ethics Committee, the Ugandan National Council of Science and Technology, the University of KwaZulu-Natal Biomedical Research Ethics Committee, Griffith University, and the Mass General Brigham Human Research Committee.

## Results

Out of 877 participants screened for eligibility in the REVAMP trial, 37 did not meet the inclusion criteria and 840 participants were enrolled in the study [28]. Half of participants were from South Africa (n = 420) and half from Uganda (n = 420), Table 1. Mean age was 38 years in South Africa and 39 years in Uganda with more males in South Africa (54% vs. 43%). Fewer participants in South Africa had VL ≤50,000 copies/mL (70% vs. 75%). The study population in South Africa had more participants with a CD4 count < 200 cells/µL (45% vs. 30%), the same proportion with CD4 200–499 cells/ µL (42% vs. 41%) and fewer participants with a CD4 count ≥ 500 cells/ µL (14% vs. 29%). There were more participants with at least one comorbidity in South Africa versus Uganda (40% vs. 26%) and at least one opportunistic infection (51% vs. 16%). Fewer participants reported side effects (14% vs. 16%) and significantly more participants reported 100% adherence in South Africa (37% vs. 22%). The mean EQ-5 L-3L at baseline was higher in South Africa compared to Uganda (0.894 vs. 0.830).

**Table 1:** Baseline Characteristics for South Africa and Uganda for individuals failing first line antiretroviral therapies (viral load > 1,000 copies/mL)

Country	South Africa	Uganda	p-value
Sample size (n)	420	420	
Age (years) (mean, SD)	38.1 (9.4)	38.5 (11.0)	0.558
Males (n, %)	228 (54.3%)	182 (43.3%)	**0.001**
Viral load (copies/1,000 mm^3^) (n, %)			
≤ 50,000	294 (70.0%)	316 (75.2%)	0.089
> 50,000	126 (30.0%)	104 (24.8%)	
CD4 count (cells per mm^3^) (n, %)			
<200	186 (44.8%)	108 (29.7%)	**< 0.001**
200–499	173 (41.7%)	150 (41.2%)	
≥500	56 (13.5%)	106 (29.1%)	
missing	5	56	
Comorbidities (n, %)			
Yes	169 (40.4%)	106 (25.7%)	**< 0.001**
No	249 (59.6%)	306 (74.3%)	
missing	2	8	
Opportunistic infection (n, %)			
Yes	214 (51.0%)	67 (16.1%)	**< 0.001**
No	206 (49.0%)	349 (83.9%)	
missing	0	4	
Side effects (n, %)			
Yes	57 (13.6%)	66 (15.7%)	0.388
No	362 (86.4%)	354 (84.3%)	
missing	1	0	
Adherence (n, %)			
Yes	157 (37.4%)	91 (21.7%)	**< 0.001**
No	263 (62.6%)	328 (78.3%)	
missing	0	1	
EQ-5D-3 L utility			
Median (IQR)	1 (0.146)	1 (0.402)	**< 0.001**
Mean (SD)	0.894 (0.175)	0.830 (0.214)	**< 0.001**

ART=antiretroviral therapy; CI=confidence interval; n= number; SD = standard deviation

Results of the two-part regression model are shown in Table 2.

**Table 2:** Two-part model of association between determinants and EQ-5D-3L health state values in South Africa and Uganda

Variable	Part 1: Logistic Regression EQ-5L-3L = 1			Part 2: Generalized Linear Model, EQ-5L-3L < 1		
	Odds ratio	95% CI	p-value	Co-efficient	95% CI	p-value
South Africa						
Male (ref female)	1.85	**1.19, 2.87**	**0.006**	-	-	-
Comorbidities (ref no CM)	0.29	**0.18, 0.46**	**< 0.001**	-	-	-
HIV treatment side effects (ref no SE)	0.39	**0.20, 0.75**	**0.005**	-0.08	**-0.14, -0.02**	**0.008**
Opportunistic infection (ref no OI)	-	-	-	-0.10	**-0.15, -0.05**	**< 0.001**
Viral load > 50,000 (ref VL≤50,000)	-	-	-	-0.06	**-0.11, -0.01**	**0.029**
100% Adherence	-	-	-	0.07	**0.02, 0.13**	**0.007**
Uganda						
Male (ref female)	1.87	**1.24, 2.84**	**0.003**	-	-	-
Comorbidities (ref no CM)	0.56	**0.35, 0.90**	**0.016**	-	-	-
Opportunistic infection (ref no OI)	0.41	**0.23, 0.72**	**0.002**	-	-	-
100% Adherence (ref < 100%)	1.64	**1.00, 2.70**	**0.049**	-	-	-
CD4						
<200 (ref)						
200–499	-	-	-	0.07	**0.01, 0.13**	**0.027**
≥ 500				0.11	**0.04, 0.17**	**0.001**

CM = comorbidities; OI = opportunistic infection; SE= side effects; ref = reference. Non−significant variables are removed from part 1 and part 2 of the model

For part I (logistic regression), in South Africa, male participants were more likely to report perfect health compared to females (OR = 1.85; 95% CI = 1.19, 2.87; p = 0.006). Participants with co-morbidities or side effects were less likely to report perfect health (OR = 0.29; 95% CI = 0.18, 0.46; p < 0.001 and OR = 0.39; 95% CI = 0.20, 0.75; p = 0.005, respectively). For part II of the model (generalised linear model), high VL, presence of opportunistic infections and experiencing ART side effects were each associated with lower HRQoL (mean difference = -0.06; 95% CI = -0.11, -0.01; p = 0.029; mean difference = -0.10; 95% CI = -0.15, -0.05; p < 0.001; mean difference = -0.08; 95% CI = -0.14, -0.02; p = 0.008 respectively). Participants with 100% ART adherence reported significantly higher HRQoL (mean difference = 0.07; 95% CI = 0.02, 0.13; p = 0.007).

**Fig. 1:**
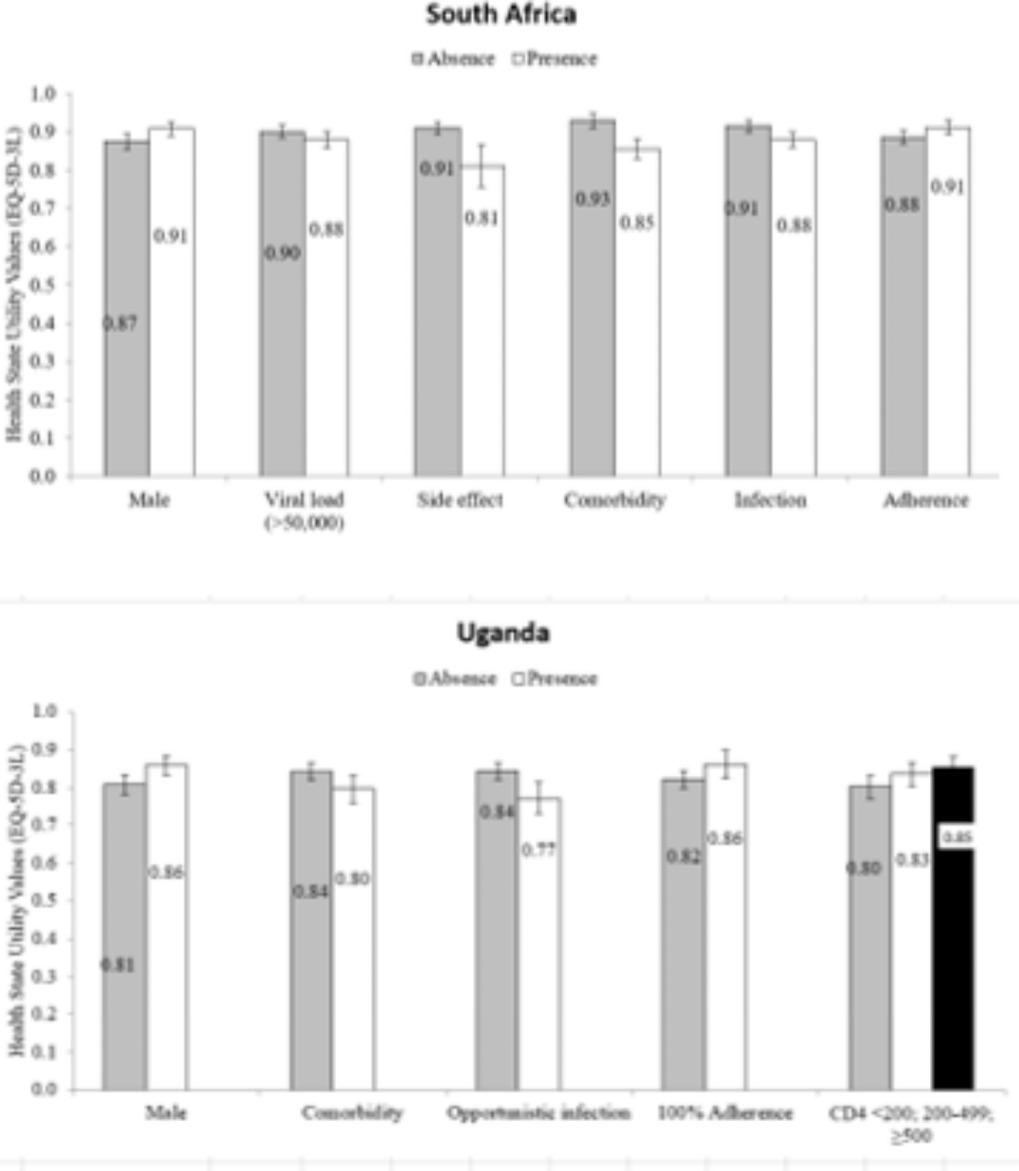
Impact of significant determinants on health state utility values (EQ-5D-3L) in South Africa and Uganda

Results were similar in Uganda where for part I, males were more likely to report perfect health (utility = 1) (OR = 1.87; 95% CI = 1.24, 2.84; p = 0.003). Participants with comorbidities or opportunistic infection were less likely to report perfect health (utility = 1) (OR = 0.56; 95% CI = 0.35, 0.90; p = 0.016 and OR = 0.41; 95% CI = 0.23, 0.72; p = 0.002). Participants with 100% treatment adherence were more likely to report perfect health (utility = 1) (OR = 1.64; 95% CI = 1.00, 2.70; p = 0.049). For part II, participants with higher CD4 count had higher HRQoL (mean difference = 0.07 for CD4 count of 200–499; 95% CI = 0.01, 0.13; p = 0.027; mean difference = 0.11 for CD4 count ≥ 500; 95% CI = 0.04, 0.17; p = 0.001) compared to participants with lower CD4 counts (CD4 < 200). The impact of significant determinants on the health state utility values (EQ-5D-3L) for South Africa and Uganda are shown in Fig. 1.

As sensitivity analysis, we compared the two-part regression model with Tobit Regression and Ordinary Least Squares, which are common alternative methods for analysing health-related quality of life. Results confirmed that in South Africa, gender, comorbidities, and side effects and in Uganda gender, comorbidities, adherence, opportunistic infection remained significant determinants of HRQoL as shown in Supplement Table 1.

## Discussion

This is among the first studies to investigate determinants of HRQoL in a population of PWH experiencing virological failure. We found numerous features of HIV disease control, including the presence of opportunistic infections, the presence of side effects and lower CD4 count were strongly associated with HRQoL. In addition, we found that men were more likely to report perfect health in this population. Finally, we found that people with the highest adherence also had higher HRQoL. Taken as a whole, these findings confirm the importance of effective HIV care in improving quality of life for PWH, and highlight key populations, such as women and those with poor adherence and virologic failure, that might be particularly key targets for intervention.

Our first key finding was that poor disease control and markers of advanced HIV: namely high VL in South Africa, low CD4 count in Uganda, and opportunistic infection in both countries, were associated with lower HRQoL. These findings agreed with other studies on viral load [12, 29], CD4 count [11, 12, 15, 29] and opportunistic infection in LMIC [11, 12]. However, these findings contradict Mutabazi-Mwesigire’s prospective study designed specifically to explore the relationship between CD4 and HRQoL in Uganda [30]. Controlling for sociodemographic characteristics, clinical and behavioural factors, the study found no association between change in CD4 count and quality of life scores by univariate and multivariate analyses [30]. This is possibly because the primary determinant was change in CD4 count rather than CD4 count categories per se. Another potential explanation could be the CD4 count thresholds that were applied: <100; 101–350 and > 350 which do not correspond to the WHO thresholds < 350 (stage 3 advanced HIV); 350–499 (stage 2 HIV infection); ≥500 (stage 1 HIV infection) [30]. The threshold of 500 (rather than 350) seems to represent the threshold for “well” versus “unwell” and therefore may better capture a difference in HRQoL. Another possible reason could be due to the instruments used to measure HRQoL which were the Medical Outcomes Study HIV health survey and the Global Person Generated Index [30]. Finally, it may depend on the point in time when PWH are surveyed. Even PWH with advanced disease may feel a return to health as soon as 6 to 12 months after starting ART. VL is an important clinical indicator of ART adherence ART response, our study findings enforce that the early detection of virological failure and achievement of virological suppression are critical to improving HRQoL in this population [31]. Furthermore, significant positive associations between CD4 and utility support the importance of CD4 count monitoring in clinical settings in Uganda.

Our second key finding was that gender is a major correlate of HRQoL among PWH failing treatment in sub-Saharan Africa. Other studies of PWH from LMIC have also reported a significant association between female gender and lower EQ-5 L-3L (South Africa, Pakistan, Colombia, Vietnam) [11, 12, 15, 29]. This finding contrasts a large body of evidence suggesting that women have received the main benefits of HIV care in the region [32, 33]. One hypothesis is that men who do link to care are a unique, self-selected healthier group of PWH and that this body of evidence thus is biased towards men with relatively higher HRQoL.

We also found that side effects and comorbidities were associated with significantly worse HRQoL. This is in keeping with Mwesiga, who found that participants with depression and pain comorbidity in Uganda rated their HRQoL as poor in all domains of the WHO-BREF [34]. Belay and Nigusso both reported an association between comorbidities and reduced HRQoL in Ethiopia using EQ-5D-5L and PROMIS Global 10 respectively [35, 36]. Two other studies found a non-significant association between side effects and lower quality of life: Bhargava (EQ-5D-3L South Africa) and Kauf (SF-6D multi-site). Non-significant findings may have been due to the low proportion of side effects in the studies (6% and 12% respectively) [29, 37]. Furthermore part 1 of our two-part model showed that in South Africa and Uganda participants with comorbidities were less likely to report perfect health. These findings suggest that good control of co-morbidities can significantly impact on HRQoL and health care systems should not overlook the treatment and monitoring of comorbid conditions. The association between side effects and lower utility may be averted in the case of newer treatment options.

A fourth key finding was that 100% adherence was associated with significantly better HRQoL in both countries. One other study looked at the relationship between adherence and HRQoL and showed that participants reporting 100% adherence achieved significantly higher HRQoL scores compared to poorer adherers which was in keeping with our findings [38].

Treatment duration and age were not significant factors in our models. Tran showed that longer duration on ART was associated with higher EQ-5D-3L [11]. Ahmed also found age to be non-significant for 12–48 months and significant for > 48 months [12]. Another LMIC study using EQ-5D-3L in South Africa reported a non-significant trend for higher age and higher EQ-5D-3L [29]. However, other studies have found that older age was associated with significantly poorer HRQoL including Mokgethi using the AIDS Clinical Trial Group questionnaire in South Africa; Ahmed using EQ-5D-3L in Pakistan and Belay using EQ-5D-5L in Ethiopia [12, 13, 35]. One potential explanation could be due to confounding, since the studies include participants with virological failure and virological suppression [12, 35]. Alternately it may be that the effect of older age is captured in other related factors such as higher comorbidities, and that the studies that reported significant age effect may not include these related factors. If age was a determinant of HRQoL then this would suggest that healthcare systems should better support older individuals with HIV as their HRQoL would be disproportionately affected, however our study did not support this hypothesis.

The interpretation of our results should be considered in the context of the study limitations. This is an analysis of cross-sectional data therefore association but not causation can be demonstrated. There were slight differences in across country results and the reasons for this are not clear. We did not collect data on smoking status, education level, employment as included in other published models and this may account for discrepancies between findings. For example, food access, diet quality, income, education, and employment have been shown to be associated with better HRQoL in Uganda and Zimbabwe [39, 40]. The valuation of health state utilities is based on reference values from Zimbabwe because no dataset is available for South Africa or Uganda. The ceiling effect of the EQ-5D-3L is widely reported and this was confirmed in our study where our median was one and more than 50% of our baseline participants reported full health. Better results may have been possible if the EQ-5D-5L version had been used [41]. Since EQ-5D-3L is a generic quality of life instrument, it may not be as sensitive to changes in quality of life as disease specific instruments. Comorbidities, opportunistic infections, and adherence were self-reported and reviewed in medical records.

Finally, our study was conducted prior to the availability of integrase strand transfer inhibitors with 72% of our participants receiving a regimen of tenofovir, emtricitabine, and efavirenz [28]. More research is required to understand the factors affecting the reporting of full health by the EQ-5D-3L. Although a ceiling effect is well-reported it is not clear why males are more likely to report full health. Also, there is a biological and clinical relationship between VL and CD4 count, and this relationship has not yet been adequately captured in regression models published to date. It is interesting to note that VL was a significant determinant in South Africa and CD4 count was a significant determinant in Uganda so additional research would be required to understand the reasons for this between country difference.

These findings are important for informing clinical care. Our key findings confirm that markers of advanced disease in addition to side effects and comorbidities were associated with a significant impact on HRQoL. Comorbidities, opportunistic infections, side effects, CD4 count and viral load are all indicators of “wellness” and ongoing monitoring of these clinical variables is required to maximise quality of life. Treatment approaches that monitor viral load and CD4 count continue to be important.

## Conclusion

Markers of advanced disease (opportunistic infection, high viral load, low CD4), side effects, comorbidities and lack of ART adherence negatively impacted HRQoL for PWH experiencing virological failure.

### Electronic supplementary material

Below is the link to the electronic supplementary material.

**Supplement Table 1**: Tobit and Ordinary Least Squares Regression of association between determinants and EQ-5D-3L health state EQ-5L-3L values.

## Data Availability

The datasets used and/or analysed during the current study are available from the corresponding author on reasonable request.
