# Supplementary material for: Determinants of health-related quality of life in people with Human Immunodeficiency Virus, failing first-line treatment in Africa
Source: Health Qual Life Outcomes. 2023 Aug 21;21:94. doi: 10.1186/s12955-023-02179-x (PMC10441724; doi:10.1186/s12955-023-02179-x)
Supplement: Supplementary file 1 — Supplement Table 1: Tobit and Ordinary Least Squares Regression of association between determinants and EQ-5D-3L health state EQ-5L-3L values. [file 12955_2023_2179_MOESM1_ESM.docx]

**Supplementary File**

**Title**

Determinants of Health-Related Quality of Life in People with Human Immunodeficiency Virus, Failing First-Line Treatment in Africa.

**Authors**

Tamlyn A. Rautenberg PhD^1,2,3^, Shu Kay Ng PhD^1,2^, Gavin George PhD^4,5^, Mahomed-Yunus S. Moosa MBChB^6^ Suzanne M. McCluskey MD^7,8^, Rebecca F. Gilbert BA^7^, Selvan Pillay, MSc^6^, Isaac Aturinda MBA^9^, Kevin L. Ard MD^7,8^, Winnie R. Muyindike MMed^9^, Nicholas Musinguzi MS^9^, Godfrey Masette BSc^9^, Melendhran Pillay MSc^11^, Pravi Moodley MMed^7,11^, Jaysingh Brijkumar MBBS^6^, Rajesh T. Gandhi MD^7,8^, Brent Johnson PhD^12^, Henry Sunpath MBChB^6^, Mwebesa B. Bwana MBChB^9^^, Vincent C. Marconi MD^13,14^, Mark J. Siedner MD^6,7,8,9,10^

^1^Centre for Applied Health Economics, Griffith University, Brisbane, Queensland, Australia

^2^Menzies Health Institute Queensland, Australia

^3^Metro North Hospital and Health Service Queensland, Australia

^4^Health Economics and HIV Research Division, University of KwaZulu-Natal, Durban, South Africa

^5^Division of Social Medicine and Global Health, Lund University, Lund, Sweden

^6^College of Health Sciences, University of KwaZulu-Natal, Durban, South Africa

^7^Department of Medicine, Massachusetts General Hospital, Boston, Massachusetts, United States

^8^Department of Medicine, Harvard Medical School, Boston, Massachusetts, United States

^9^Faculty of Medicine, Mbarara University of Science and Technology, Mbarara, Uganda

^10^Africa Health Research Institute, KwaZulu-Natal, South Africa

^11^National Health Laboratory Service, Durban, South Africa

^12^Department of Biostatistics and Computation Biology, University of Rochester, Rochester, New York, United States

^13^Department of Medicine, Emory University School of Medicine, Atlanta, Georgia, United States

^14^Department of Global Health, Rollins School of Public Health, Atlanta, Georgia, United States

^ Deceased.

**Sensitivity analysis**

We compared the two-part regression model with Tobit Regression and Ordinary Least Squares, which are common alternative methods for analysing health-related quality of life, as shown in Supplement Table 1.

Supplement Table 1: Tobit and Ordinary Least Squares Regression of association between determinants and EQ-5D-3L health state EQ-5L-3L values.

| Analysis | Tobit Regression | | | Ordinary Least Squares Regression | | |
| --- | --- | --- | --- | --- | --- | --- |
| Variable | Coefficient | 95% CI | p-value | Coefficient | 95% CI | p-value |
| South Africa (n=420) | | | | | | |
| Male | 0.16 | **0.07, 0.26** | **0.001** | - | - | - |
| Viral load >50,000 | 0.04 | -0.08, 0.17 | 0.505 | **-0.04** | **-0.00, -0.07** | **0.033** |
| VL#male | -0.21 | **-0.38, -0.04** | **0.015** | - | - | - |
| Comorbidities | -0.29 | **-0.38, -0.20** | **<0.001** | -0.11 | **-0.15, -0.07** | **<0.001** |
| Side effects | -0.72 | **-1.06, -0.39** | **<0.001** | -0.40 | **-0.56, -0.24** | **<0.001** |
| SE#CM | 0.61 | **0.26, 0.96** | **0.001** | 0.34 | **0.17, 0.50** | **<0.001** |
| Uganda (n=420) | | | | | | |
| Male | 0.14 | **0.06, 0.23** | **0.001** | 0.06 | **0.02, 0.11** | **0.006** |
| CD4 200-499 | 0.03 | -0.06, 0.13 | 0.502 | 0.03 | -0.02, 0.08 | 0.287 |
| CD4 ≥ 500 | 0.10 | -0.01, 0.21 | 0.082 | 0.06 | **0.01, 0.12** | **0.037** |
| CM | -0.14 | **-0.23, -0.05** | **0.003** | -0.08 | **-0.13, -0.03** | **0.001** |
| OI | -0.16 | **-0.27, -0.06** | **0.003** | -0.09 | **-0.15, -0.03** | **0.003** |
| Adherence | 0.11 | **0.01, 0.21** | **0.040** | - | - | - |

# = Interaction; VL=viral load > 50,000 copies per 1,000 mm^3^, OI opportunistic infection; SE= side effects; CM = comorbidities.
